# The Heart Clot—Its Cause

**Published:** 1849-07

**Authors:** Charles D. Meigs

**Affiliations:** Professor of Obstetrics in the Jefferson Medical College, Philadelphia


*The Heart-Clot—its Cause.* By CHARLES D. MEIGS, M.D., Professor of Obstetrics in the Jefferson Medical College, Philadelphia.

I beg leave, through the columns of your useful periodical, to present the statement of certain opinions I have long entertained, relative to points in pathogeny connected with the occurrence of endo-cardial coagula; and I do so because I consider them deserving of serious consideration by the practitioner.

These opinions are connected with certain points of practice or treatment that are, in many cases, indispensably necessary for the safety of the sick; and my sole desire in offering the communication, is founded on the hope that it may tend to prevent some disastrous events, which the want of a little reflection might allow.

I believe it is a fact, not to be controverted, that in an animal slowly bled to death, the first portions of blood extravasated, coagulate less readily than the last portions. If this doctrine is true, it follows that the coagulability of the blood left in the vessels after great hemorrhages is augmented: I have had several occasions to find that it is dangerously augmented.

To take one of the most ordinary cases of hemorrhage —I mean that occurring after labor, or in abortion—we have an instance in which, even after the arrest of the bleeding, the patient is exposed to mishap from the coagulability of the blood remaining in the vessels. Loss of blood produces a tendency to fainting, or lipothymia: during an attack of fainting, the motions of the heart are enfeebled, the diastole slow—torpid, for the blood moves languidly in both the vena cava, pours itself out in a slow current into the auricle, which it sluggishly distends, and sometimes is then instantly converted into a solid clot. If a clot be formed in the right auricle, it will also be formed in the *iter ad ventriculum dextrum* filling up the cone of the tricuspid valve; and the nucleus of it will cause the coagulum at length to occupy the cavity of the right ventricle, and extend itself to a greater or less distance along the tractus of the pulmonary artery. If the whole pulmonic side of the heart should be perfectly occupied in this way, the death of the individual would be instantaneous; and I doubt not, that many of the examples of sudden death, after delivery in hemorrhagic labors, are produced by the formation of cardio-morphous coagula which form in the instant of a state of fainting, or lipothymia. It is understood that the young Princess Charlotte, whose death at Clermont cast a mournful gloom over the whole British Empire, died within fifteen minutes after the birth of the princess, and that there was no very considerable hemorrhage, no laceration, nor other incident that might fitly explain the suddenness of her decease. Many women are known to perish in this manner. I have been the eye-witness of instances of the kind. I have also seen a very great number of persons, who appeared to me to be in danger of perishing in the same way, but who escaped a fate so deplorable. I am aware also of istances in which women, after considerable hemorrhagic losses, have been esteemed by their physicians to be what is called doing well, during a space of from one to seven days, but who afterwards becoming *instantly* extremely ill, have perished without remedy in from two to twenty days thereafter.

If a surgeon, desirous to reduce a luxated humerus, should attempt to do so, he might find the resistance of the muscular contraction so great as to prevent his success, and he would therefore probably resolve to take away the resistance of the muscular contraction, by bleeding his patient *ad deliquium.* The surgeon knows that the deliquium would take effect upon the loss of a much smaller quantity of blood if the patient should be placed upon his feet in a standing posture, than if he were to recline upon his bed in a low recumbency. He would bleed the man while in an erect position. This ordinary practice is conformable with the dictates of experience in all cases of fainting, for it is well known that an individual will faint more readily in a vertical, than in a horizontal position; and the first idea that is obvious to any medical man in a case of fainting is this —that he shall cause the patient to be laid with the head very low, taking away for the time even the pillow. I have, on many occasions, besides taking away the pillow, found myself under the necessity of elevating the foot of the bed by placing books or blocks under the lower bed-posts in order to favor the determination of blood to the encephalon; for I conceive that in all cases of fainting the brain has become oligemic.

I may assert the opinion here, that fainting is oligemia of the encephalon, and that a hyperemia of the encephalic bulbs is the very converse of, and absolutely incompatible with the state of swooning. To raise up a woman who has within the few days past lost a considerable quantity of blood is almost inevitably to bring on deliquium. Now, if the idea be just that hemorrhage renders the remaining blood more coagulable, then it follows, that to take the woman out of bed, or to let her sit up in bed, is to expose her to the hazard of forming a coagulum in the right auricle, which, by extension of the nucleus, may fill the ventricle, occupy the aperture of the tricuspid, and pass several inches upwards in the course of the pulmonary artery and its branches. Monthly nurses, and the ordinary attendants of the sick know nothing of these things, and they hesitate not oft-times, to exhort or to permit the anemical accouchée to rise and sit for a few moments for purposes that might be answered without quitting the horizontal position.

A lady was taken in labor in the afternoon. She sat in her arm chair all night without sleeping: at 5 o'clock in the morning she placed herself upon the bed and the child was born in half an hour. The placenta was spontaneously and perfectly extruded, nothing being left in the womb: it was her fifth labor. Within an hour she had hemorrhage—the vagina and uterus contained large coagula which were turned out by the physician, whereupon the hemorrhage ceased; she may have lost altogether some thirty ounces of blood. He remained near her for several hours. At mid-day, throughout the afternoon, and during the following night, she appeared to be perfectly well. At half past nine the following morning the physician made his visit; she was without pain or the least indisposition, nor had she any symptoms, save those that appertain to the condition of a healthy accouchée. Her pulse was about 75 beats per minute; the respiration, temperature, and hue, satisfactory to the medical attendant; her complacency, physical and moral, was absolute.

The physician left her at 10 o'clock in the morning. Being summoned again, he reached her apartment at 1, P.M., and found her in a state, which led him to suppose that she might be near dying. The pulse was 164 per minute, very feeble, and thread-like; the hands were cold, and the respiration was performed apparently by the strong effort of her will only. The respiratory acts were performed with great violence, and without rythm. Auscultation of the heart disclosed a feeble impulse, with great irregularity of the systolic action. She had lost no more blood beyond the ordinary lochial discharge; the vagina, which was examined, contained no coagulum.

When I came into the apartment at 3 o'clock, P.M., she supposed herself to be in a dying state and asked me if I thought she would live half an hour. It is difficult to conceive of a spectacle of more extreme physical distress than that presented by this dying lady. Every respirtory act was attended with violent pain referred to a place near the lower extremity of the sternum, as in angina pectoris. Palpation of the abdomen and questions relative thereto, showed nothing abnormal there. Upon retiring for consultation, I expressed to my medical brother the opinion that the pulmonary heart was filled with a coagulum or false polypus; the prognostic therefore, was necessarily fatal.

She had been left at 10 o'clock in the morning with a pulse at 75, and in the course of the forenoon she had been taken up from her recumbent position, and allowed to sit upon the close-stool for the purpose of evacuating the bladder of urine, immediately after which she was ill, and the physician sent for.

I made this diagnostic upon these grounds, viz: I said there is no pathogenical principle that I know of that can explain the change of her pulse from 75 to 164, in so short a time, save that of a mechanical obstruction formed by a clot or tampon filling up the cavities of the heart. It is clear that there is no scarlatina, no variola, no fever of any kind—no attack of Asiatic cholera nor other malady, that is capable of making, so soon, so great a change in the action of the heart as is here observed. The patient had hemorrhage yesterday, which has increased the coagulability of her blood; she was taken out of her recumbent position and placed upright in bed, whereupon she became suddenly ill in consequence of the coagulation of blood in her auricle, and there is no power that is able to remove this tampon from the cavity of her heart; it will destroy her as effectually as would a musket-ball deposited in the ventricle.

The respiration in this case was carried on at the time of my arrival, solely by the force of the voluntary power. There seemed to be no rythmical respiration whatever; when she ceased to breathe by her volition, her respiration appeared to be suspended altogether. As might be expected, these voluntary aspirations were not rythmical, but interrupted, uncertain, having long intervals. The blood that came up from the inferior cava and down from the upper cava, must have passed with great difficulty between the superficies of the clot, and the paries of the heart. It must have moved in small quantities only through the tricuspid, and when distending the pulmonary ventricle, that ventricle could contain but a small portion of fluid blood, being mainly occupied by the coagulum. A similar difficulty existed as to the efflux of the blood along the pulmonary artery, which was tamponed at the time with a cylindrical clot extending several inches along the vessel and its principal branches. Under these circumstances, the quantity of carboniferous blood entering the lungs by the the pulmonary artery, for aeration, could be a small quantity only; hence the violent almost spasmodic protracted efforts to aspire the air of the atmosphere; efforts which, however great, must measurably fail of the purpose of abolishing the direful sense of pulmonary oppression, or respiratory distress, or to use a more concise term, asphyxiation. The quantity of blood in the lungs was too small to receive the endowment of oxygen which is requisite to preserve any individual from a feeling of suffocation; and however thorough might have been the aeration of the small quantity that was there, however brilliant and florid may have been its arterial hue after being breathed upon, the quantity of oxygen imparted to it must necessarily be insufficient so to act upon the nervous mass, the neurine, as to hinder the conscious principle from perceiving the sense of asphyxiation. With a heart situated in this manner—with the utter impossibility of thoroughly oxygenating the sanguine mass, the innervation gradually fails—a failure which is manifested in the decadence and ultimate overthrow of the various functions. All the functions are but the expressions of the biotic force that is sent down by the encephalic bulbs and spinal cord of the distal points of the nerve-fibrils in the organs. Every acinus of a gland is alive solely by the nervous force which comes into it by the fibril that connects it with the nervous mass, to obey whose mandate is to live, while to fail of receiving it is command to die; the same is true of every part and particle of the histological constitution.

As the encephalic bulbs certainly cease to irradiate the organs when they themselves cease to receive through the oxygeniferous streams injected into them by the carotids and vertebrals, the supplies of oxygen which alone enable them to evolve the life force, the nerve force, the *lebenskraft*, the biotic force—it follows, that the organs die in the same ratio as those bulbs fail and perish.

One is not surprised, therefore, upon observing that a person in good health, like this unfortunate lady, the right side of whose heart becomes suddenly, instantaneously tamponed by a coagulum, should fall a victim, and that speedily, not to the presence of the clot alone, but to disease developed in other parts, whose life is overthrown in consequence of the obstruction of the prime organ of circulation. Only a few hours could pass with a large coagulum in the heart, before the pericardium would begin to be filled with serum, or the embarrassments in the pulmonary circulation seek in vain for relief, by pouring out a vast effusion of water into the cavities of the pleura; or the innervative force being withdrawn from the viscera contained within the abdomen, whose venous blood is prevented from flowing off through the pulmonary artery, there is set in motion in the peritoneal sac, a tide of effusion filling it up in the course of a few hours.

In all such cases as those of which I am speaking, the escape of the blood from the venous side of the sanguine circle is retarded, with the effect of producing enormous engorgements of all those venous branches, which usually and readily allow their products to run off through the ascending and descending cava. Let the reader perpend for a moment the condition of that portion of the vascular system which receives aortic injections by the celiac and the superior and inferior mesenteric arteries: let him reflect that the whole of this torrent, which is entirely expended upon the chylopoietic and alimentary organs, is first collected by the capillary radicles of the portal vein, then distributed again among the capillary termini of the hepatic porta, whence it is a second time collected to flow off by the hepatic veins. Now, if the auricle and ventricle are tamponed by an endo-cardial coagulum, this whole torrent is inevitably arrested, and the cavities become immediately engorged by the continued injections from the aorta, leaving no grounds of astonishment as to the sudden or fatal derangement of the healthy states of the tissues that are developed by it.

The time required for extinguishing the life of the sufferer is a variable time; one relative to the magnitude and extent of the coagulation. I can imagine that in the case of the Princess Charlotte, already alluded to, a coagulum was formed which filled the heart so completely, as to put an end to its action within fifteen minutes after the birth of the princess. My patient, above mentioned, lived forty-eight hours after the occurrence of the accident, during which time she suffered the most inexpressible respiratory distress. She filled her pericardium with serum, while her peritoneal cavity became also the subject of a great effusion. Upon examining the heart twenty-four hours after her decease, one might feel surprised that her life could be so long protracted, since the auricle, tricuspid, and ventricle were completely tamponed with a clot which was not an enthanasial clot, but consisted apparently of a firm, whitish yellow mass of fibrin, out of which every particle of hematoglobulin had been washed away, or expressed. An enthanasial clot is, in my opinion, necessarily a red one; a prëenthanasial one ought to be white.

A patient in this city was delivered early in the morning. Soon after the birth of the child and the delivery of the placenta, the physician descended to the breakfast room, having given strict charge that the patient should preserve the recumbent position and be kept quiet. While at his breakfast, cries from the top of the stairway called him, for "God's sake," to hasten to the assistance of the patient. In a moment he was at her bedside, where he found her already dead, having fallen backwards across the bed, with her legs hanging over its side. He was told that she had said to her nurse, "I wish to get up,"—"The Doctor says, madame, you must not get up, if you please." "But I must get up, I will get up." She threw her feet out of the bed, and rose up sitting upon its edge; her head reeled to and fro, and she fell back and expired. No examination was made of the dead body, but I ask the reader to explain the cause of this sudden death, otherwise than by the rationale that her heart ceased to beat because it became instantly filled with an immovable clot.

Man cannot die, save by the cessation of the activity in the brain, or in the heart, or in the lungs: he lives within this triangle, and can only escape at one of its angles. He must die by the brain, or by the heart, or by the lungs. It is to the last degree improbable that this woman perished solely because her brain ceased to evolve; but if it did not instantly cease to evolve, it must have continued to be the cause of motion everywhere. If the heart, as I suppose, became instantly filled with congealed blood, so that it could no longer receive nor discharge any portion of that fluid, the nervous mass would cease to live as soon as it should have consumed all the oxygen contained within its capillary vessels at the moment of the arrest of the cardiac circulation. The patient died by the heart.

A lady was confined in a natural labor, giving birth to a healthy child, at term. She lost a considerable quantity of blood at the time of the extrusion of the placenta, which left her feeble and pale. Her physician directed her to be kept quiet. She had a good day, and following night. At the morning visit the physician found her comfortable, and her condition was satisfactory to him. Soon after he left her apartment she was seized with violent alarming illness, whereupon he was recalled, and was again present after the lapse of about an hour. Her pulse was extremely frequent, feeble, and small; it continued frequent until the moment of her death, which took place about the nineteenth or twentieth day. On the eighteenth day, I think, I saw the lady, and formed the opinion that she was perishing on account of a false polypus, clot, or tampon in the heart, established there by the imprudent early uprising after a hemorrhage. After her death a great quantity of water was found in the cavity of the right pleura, while a firm white coagulum, entirely destitute of corpuscles, was detected in the right auricle, filling up very much the cone of the tricuspid, while the ventricular end of it seemed to be torn or threshed to pieces by the cordæ tendineæ, which, during so many days, had been vainly occupied in the endeavor to demolish it. The filling up of the pleura with serum was a natural consequence of the condition of the respiratory organs, quite as much so but not at all more so, than was the filling up of the peritoneum and pericardium in the former case, consequences of the arrest of the circulation in the cava and its branches.

Towards the end of the year 1848, a primapara gave birth to her first child. She was tall, very slender, and delicate; the placenta was not removed; she lost a good deal of blood. Between forty and fifty hours after the birth of the child, upon being called to her succor, I removed the placenta from the cervix uteri in which it was grasped and detained. I removed it with the index finger of my right hand. The stench of it was noisome to the last degree. The putrid odor of it remained upon my hand for twenty-four hours, notwithstanding every effort to remove it. The patient was pale, and her pulse somewhat frequent, presenting the usual characteristics of the anemical pulse. On the following day she was comfortable; the milk was secreted, the lochia healthy, and she was doing well, though still very pale. On the seventh day, she was placed in a chair before the fire, sitting up: she immediately felt sick, was put to bed, and I being called to see her, told her friends that she had formed a fatal coagulum in the heart. She lived about forty-eight hours after the accident; I did not examine her body. I leave the reader to judge whether my diagnostic was or was not probably correct. She had a pulse upwards of one hundred and sixty; the impulse of the heart feeble; the respiration disturbed, frequent.

On a great many occasions since I have been a practitioner of medicine, I have been called to see patients, who, after hemorrhagic labors, have disobeyed my injunctions as to horizontal rest, and who being prematurely lifted upright in bed, had fainted. I have not a doubt that among those of these persons in whom I found the heart fluttering, irregular, and feeble in its action on my arrival, incipient coagulation existed. I have thought as I entered the room of a patient, that her auricular blood had begun to thicken, but was driven out from the auricle before its thorough coagulation, in consequence of the startling effects of a dash of cold water upon the face, or of clapping the hands, or snatching the pillow from under the head and shoulders, allowing the head to fall so as to favor the restoration of its vascular tension or even hyperæmia, and thereby reestablishing the perfect and powerful extrication of its innervative force. The re-excitation of the innervative force of the brain would probably soon enable a heart so situated to discharge itself of the inchoate coagulum.

It is not needful that I should draw out this paper to any great length; nor that I should discuss the reasons why so many autopsies present the evidences of the endocardial clot of which I have spoken, without having excited in the mind of the attendant practitioner, the suspicion of its presence before the death of the patient. It appears to me, to be enough for the present occasion, to propound the question, Can a patient with a white clot in the auricle and ventricle recover? If such a clot be a small one, the pulmonary circulation, although checked, is not necessarily suspended, but the nucleus of such a clot, like the nucleus of an urinary calculus, tends constantly to increase in size, and hence a small coagulum, which strangely disturbs the action of the heart, may consist with a considerable protraction of the struggle against its fatal power over the circulation. The gradual augmentation of the volume of the clot, and its extension into the pulmonary artery and its branches must in every case lead to an inevitable dissolution. I have not the least confidence in the power of alkaline medicines to dissolve such coagula, nor do I admit that the dull white endo-cardial coagulum so often discovered is the result of a state of endo-carditis; but I rather attribute its occurrence to a temporary stasis or near approximation to stasis during a state of fainting in an exhausted patient. Its occurrence after hemorrhagic labors, or upon the almost total suspension of the circulation at the cessation of an attack of puerperal eclampsia ought not to excite surprise. If a coagulum should fill the auricle and the tricuspid valve completely and at once, the death would be almost instantaneous and the clot would be found red. If the process of its formation should be long protracted it would be dull white.

I did not design in this paper, to speak at all of the enthanasial coagulum; it is perhaps quite normal that some portions of the blood last reaching the heart, at the moment of death, should congeal there.

In regard to the diagnosis of cases in which the endo-cardial coagulum becomes suddenly constituted, as in the examples of which I have spoken, it appears to me that the medical observer, in order to make it, must resort to a method which is only to be fitly characterized as transcendental diagnosis. It is true that the feeble impulse and almost complete suspension of the sounds of the heart, might serve as a quasi physical diagnosis of however little value.

By transcendental diagnosis I mean one made by a process of the mind, fitter to be called sentiment or conviction, than a regular ratiocinative progress.

To enter an apartment one has quitted only half an hour before, and to find a patient hopelessly ill with signs of imminent death, yet who had no serious symptoms of illness before—to find her making desperate voluntary efforts to breathe, without any signs of laryngeal or phrenic or pulmonic inflammation or accident—to see the face pale and ghastly—to observe her conscious sense of impending asphyxiation from loss of oxygen—without the leaden or iodic hue of a general cyanosis—These are the grounds of a diagnosis which may be called transcendental, one in which the consciousness of the physician informs him that a mechanical obstruction with n the heart exists, and that such an obstruction alone can give rise to the phenomena.

In all the lingering or sudden progressions of the accidental disorders supervening in endo-cardial coagulum, no purely cyanotic manifestations have met my observation.

Writers on cyanosis mostly refer the cyanotic symptoms to the backing of the carboniferous blood of the veins into the capillaries. You, Messrs. Editors, are aware that I have maintained the opinion that cyanosis is, in its essence, not blueness of the surface, but a state of the nervous mass produced by the absence of oxygen in the brain-capillaries.

The writers, and among them, perhaps in chief, Professor Rokitansky in his Pathologischen Anatomie, contend that cyanosis depends most commonly upon constriction of the oricfices of the great vessels of the heart, preventing the venous blood from escaping from the cavæ by the routes of the heart. Now, I aver that no obstructions existing in the vessels of the heart can be more complete than that depending upon a large endo-cardial clot, or tampon; and yet I venture to say that under circumstances of such kind the victim perishes without manifesting the peculiar livor or cyanotic tinge which characterizes the forms of the malady, that are connected with open foramen ovale and imperfect action of Botalli's valve. It is my clear conviction, that as long as the respiration can be carried on in endo-cardial clot, the blood, however small in quantity that reaches the lung passing along the superficies of the clot, is highly charged with oxygen. While, therefore, oxygeniferous blood continues to reach the brain, the patient, though conscious of the want of oxygen in due quantity, is in a state different from that of one who injects only carboniferous or venous blood into the neurine of the encephalon.

My intention was to speak only of the white clot, the false polypus, to show the probability of its being formed under circumstances of deliquium, in the oligæmia that follows uterine hemorrhage; and thereupon show how dutiful a thing it is on the part of the attendant physician, to issue the clearest and most precise orders as to the guidance of the hemorrhagic accouchée. I believe that a woman who has lost a very great quantity of blood, and who is prematurely taken out of her recumbent decubitus, and placed upright upon the close-stool, whether in bed or not, incurs a most dangerous risk of a miserable and premature death, from the sudden formation of a heart-clot.—*Medical Examiner.*

